# Reply to the Letter to the Editor: Should all trainees “do research”?

**DOI:** 10.1186/s13244-025-01954-2

**Published:** 2025-04-05

**Authors:** Luis Martí-Bonmatí

**Affiliations:** Biomedical Imaging Research Group, Instituto de Investigación Sanitaria La Fe, Valencia, Spain

In their Letter to the Editor, Should all trainees “do research”? [1], Professors Steve Halligan and Stuart Taylor reinforced that trainees lack both dedicated time and statistical support for research [2, 3]. They further highlight that “most medical research is poorly designed and executed” [4] and describe radiomics as “underpowered” [5] and lacking endorsement in patient management guidelines. Consequently, they advocate for “less but better research” instead of more [6]. They foresee a recommendation in which “training should focus on how to assess research quality because far more radiologists will read research papers than write them,” thereby fostering a training environment where research is pursued only by those with a “genuine passion” for it [1].

However, research is mandatory in professional radiology training. While we acknowledge the necessity of improving research quality, the role of research in radiology cannot be negatively understated. Radiology’s strength lies in its ability to integrate observational data with experimental evidence to improve patient care and advance medical knowledge. Research helps radiologists contribute to the evidence base that updates clinical guidelines and best practices. Even for those who do not actively conduct research, staying informed about current research is essential for maintaining a high standard of practice. University hospitals are academic medical centres where radiologists balance clinical work, education, and research to improve patient outcomes. Encouraging research in residency programs not only generates scientific papers but also develops the ability to critically evaluate existing literature. Unfortunately, meaningful research engagement during residency is highly challenging.

The notion that research training should focus primarily on assessing research quality rather than conducting research itself is an oversimplification. While critical appraisal skills are essential, hands-on research experience provides an irreplaceable foundation for understanding study design, methodology, and data interpretation. Passive learning alone is insufficient, as residents must engage in active research teams, learning through observation, collaboration, and significant contribution to ongoing studies.

Furthermore, although concerns regarding low-quality research are valid, all research contributes to the broader scientific discourse. Medical science is inherently experimental, and even studies with limited immediate clinical impact lay the groundwork for future discoveries. Rather than discouraging research in areas such as imaging biomarkers due to low clinical adoption, efforts should be directed toward improving research quality through structured training and mentorship.

In my understanding, a key distinction between leadership-driven and collaborative research is essential. Historically, radiologists have played a secondary role, providing imaging expertise in physician-led projects. This must change and radiologists should lead multidisciplinary projects instead of merely supporting research in other disciplines. I encourage mentors and senior faculty members to instil this leadership spirit in trainees, fostering a research culture. Achieving this goal is complex and requires addressing multiple factors. Beyond a natural inclination toward research, exposure to research methodologies is necessary. Residency programs must create environments that foster research engagement by incorporating structured methodologies. Two key competencies emerge as crucial for radiology trainees. First, in an era of exponentially increasing medical data, radiologists must be equipped with the critical skills to distinguish high-quality evidence from poorly conducted studies. Second, initial participation in collaborative research can serve as a stepping stone toward assuming leadership roles in research projects.

While the authors argue that a lack of allocated time and statistical support are primary obstacles to resident research participation, the broader issue lies in the absence of a structured research culture linking the technological advancements in medical imaging, the vast amounts of data, and the focus on clinically relevant research questions. Mentors play a critical role in shaping their research orientation, ensuring their efforts are impactful.

Instead of restricting research to a select few, a more constructive approach would be to expose all trainees to the basics of research early in their training; encourage mentorship and multidisciplinary teamwork over solitary research efforts; foster enthusiasm for research, recognise its cumulative value; and promote skill development in critical appraisal, statistical literacy, and research methodology. High-impact studies do not emerge in a vacuum; they require a thriving research community where ideas are tested, refined, and expanded upon by multiple contributors. If the goal is to produce “less but better research,” a critical mass of research activity must first exist. Discouraging research participation among residents due to quality concerns may inadvertently stifle future innovations.

Radiology has a relatively low number of active researchers compared to other medical specialities. The field’s limited engagement in research reduces its influence on medical innovation, clinical guidelines, and funding allocation. Given the increasing role of imaging in AI-driven diagnostics, biomarker discovery, and personalized medicine, shouldn’t this change? By expanding research exposure, we can increase the probability of developing future leaders who will drive medical imaging advancements. Just as nations invest in broad education to cultivate future scientists and innovators, radiology must foster a culture where research is more accessible, ensuring that, over time, more specialists contribute to its evolution.

In summary, a positive message and structured support are essential for cultivating a competent generation of clinician-scientists in radiology. Research paves the future of radiology, and trainees should be continuously trained according to their capabilities, understanding, willingness, and engagement. Even in high-workload environments, radiologists should actively participate in research to the fullest extent of their expertise. Developing a research culture within radiology departments should not be seen as optional, but as a survival goal for the specialty.

## Data Availability

Not applicable.

## References

[1] Halligan S, Taylor S (2025) Letter to the Editor: Should all trainees “do research”? Insights Imaging. 10.1186/s13244-025-01940-8 10.1186/s13244-025-01940-8 PMC11971067 40185912

[2] Klontzas ME, Reim M, Afat S et al (2024) Research training during radiology residency: findings from the ESR Radiology Trainee Forum survey. Insights Imaging 15:236 39373762 10.1186/s13244-024-01812-7 PMC11458849

[3] Campbell SZ, Kuah JY, Hall-Craggs M, Parry T, Mallett S, Halligan S (2022) Access to statistical support for medical imaging research: questionnaire survey of UK radiology trainees. Clin Radiol 77:920–924 36175257 10.1016/j.crad.2022.08.136

[4] Ioannidis JP (2005) Why most published research findings are false. PLoS Med 2:e124 16060722 10.1371/journal.pmed.0020124 PMC1182327

[5] Chalkidou A, O’Doherty MJ, Marsden PK (2015) False discovery rates in PET and CT studies with texture features: a systematic review. PLoS One 10:e0124165 25938522 10.1371/journal.pone.0124165 PMC4418696

[6] Altman DG (1994) The scandal of poor medical research. BMJ 308:283–284 8124111 10.1136/bmj.308.6924.283 PMC2539276

